# Distinct characteristics on mixed infection of SARS-CoV-2 variants and other respiratory pathogens among patients with acute COVID-19 in central China

**DOI:** 10.3389/fcimb.2026.1653022

**Published:** 2026-03-18

**Authors:** Yiman Geng, Youhua Yuan, Xuhong Lin, Jingjing Wei, Qi Zhang, Xiaohuan Mao, Xiaohuan Zhang, Xiulei Zhang, Yuan Zhang, Jing Zhao, Fengxia Guo, Peiming Zheng

**Affiliations:** 1 Department of Polymerase Chain Reaction (PCR), Henan Provincial People’s Hospital, Zhengzhou University People’s Hospital, Henan University People’s Hospital, Zhengzhou, Henan, China; 2 Department of Special Laboratory, Henan Provincial People’s Hospital, Zhengzhou University People’s Hospital, Henan University People’s Hospital, Zhengzhou, Henan, China; 3 Department of Laboratory, Huaihe Hospital of Henan University, Kaifeng, Henan, China; 4 Department of Neurology, Jiaozuo Municipal People’s Hospital, Jiaozuo, Henan, China; 5 Department of Clinical Microbiology, Henan Provincial People’s Hospital, Zhengzhou University People’s Hospital, Henan University People’s Hospital, Zhengzhou, Henan, China; 6 Department of Microbiome, Henan Provincial People’s Hospital, Zhengzhou University People’s Hospital, Henan University People’s Hospital, Zhengzhou, Henan, China; 7 Department of Laboratory, Henan Provincial People’s Hospital, Zhengzhou University People’s Hospital, Henan University People’s Hospital, Zhengzhou, Henan, China

**Keywords:** clinical characteristics, COVID-19, high-throughput nucleotide sequencing, mixed infection, nested PCR, SARS-COV-2 variants

## Abstract

**Background:**

Reports on mixed infection with different severe acute respiratory syndrome coronavirus 2 variants and other respiratory pathogens in patients with acute coronavirus disease in China remain scarce. In this study, we analyzed the clinical characteristics of mixed infections involving different severe acute respiratory syndrome coronavirus 2 variants and other respiratory pathogens in patients with acute coronavirus disease in central China.

**Methods:**

Nested polymerase chain reactions and metagenomic next-generation sequencing were employed to identify severe acute respiratory syndrome coronavirus 2 variants. Clinical data, including hospitalization days, severity classification, outcomes, and laboratory data, were collected and analyzed.

**Results:**

Seven patients had mixed infections with different severe acute respiratory syndrome coronavirus 2 variants in samples collected on different dates. Overall, 54.6% (83/152) of patients had co-existing respiratory pathogen infection. The most common co-existing respiratory pathogen was *Mycoplasma pneumoniae*. Longer hospital stays, intensive care unit admission, and prolonged duration from admission to positive severe acute respiratory syndrome coronavirus 2 sample detection were independent risk factors for acute coronavirus disease infection with different respiratory pathogens. Severity classification, mixed infection, cerebral fraction, and fever were independent risk factors for failed treatment. Early detection of white blood cell count, procalcitonin, and D-dimer concentrations can help predict mixed respiratory infections and treatment outcomes.

**Conclusions:**

The phenomenon of mixed infection with different variants in patients with coronavirus disease may have been underestimated. Therefore, active surveillance of severe acute respiratory syndrome coronavirus 2 variants should be performed in older patients with comorbidities.

## 1 Introduction

Coronavirus disease (COVID-19) is an emerging infectious disease identified in 2019, caused by severe acute respiratory syndrome coronavirus 2 (SARS-CoV-2) (Zavaleta, 2020). SARS-CoV-2 is an RNA virus that possesses a proofreading mechanism mediated by nsp14 exoribonuclease (ExoN), which can reduce the mutation rate; however, this proofreading capability is very limited (Moeller et al., 2022; Broni and Miller, 2023). Therefore, it leads to the production of many variants (Bouhaddou et al., 2023). The most prevalent SARS-CoV-2 variants in China were the XDV.1 and JN.1 series from June 2024 to February 2025 (Peng et al., 2024; Wu et al., 2024). For respiratory infectious diseases, such as influenza, the co-existence of various pathogens is a common phenomenon. Common risk factors for co-existing infections in patients with respiratory infectious diseases include older age, complex underlying conditions, and longer hospitalization duration (Daniels et al., 2021; Deen, 2023). Although researchers from Australia, Italy, and other nations have reported mixed infections of different SARS-CoV-2 variants (Liu et al., 2023; Ng et al., 2024; Rockett et al., 2022; Russell et al., 2024; Wawina-Bokalanga et al., 2022), limited reports regarding the types of co-existing pathogens, clinical characteristics, or outcomes of patients with acute COVID-19 and respiratory-mixed infection were available. Furthermore, to the best of our knowledge, no reports exist on mixed infections of different SARS-CoV-2 variants in patients with acute COVID-19 in China. We believe that the characteristics of SARS-CoV-2 variants prevalent in China, regarding their transmissibility, pathogenicity, and immune evasion capabilities, undoubtedly increase the complexity of co-existing pathogens and mixed infections. These variants may affect host immune responses and even alter their interaction patterns with co-existing pathogens, thereby posing new challenges to clinical manifestations, diagnosis, and treatment strategies. Therefore, conducting in-depth research on mixed infections within the current context of circulating variants is of critical importance for developing more precise public health strategies and clinical management measures, which also serves as the starting point for this study. Therefore, in this study, we aimed to analyze the clinical characteristics of mixed infections involving different SARS-CoV-2 variants and other respiratory pathogens in patients with acute COVID-19 in central China.

## 2 Materials and methods

### 2.1 Aim, design, and setting of the study

In this study, we aimed to analyze the clinical characteristics of mixed infections involving different SARS-CoV-2 variants and other respiratory pathogens in patients with acute COVID-19 in central China. Participants’ samples were obtained from the PCR department of the Henan Provincial People’s Hospital. Other respiratory pathogens included bacteria, viruses, mycoplasma and fungi. Information on patients’ infections with other pathogens is obtained from the hospital’s medical record information system. The identification of *mycoplasma pneumoniae* is carried out using immunological antibody methods, bacterial identification is done using bacterial culture and identification card, and the identification of viruses and fungi is conducted to bronchoalveolar lavage fluid by using metagenome next-generation sequencing.

### 2.2 Sample collection

Overall, 172 throat swab samples were collected from 152 patients with confirmed COVID-19 between July 1, 2024, and February 1, 2025. This was because some patients provided more than once sample during their hospital stay. We conducted SARS-CoV-2 variant identification on all these sample*s*, However, during our analysis, we classified and analyzed the data based on the 152 patients, thus excluding the duplicate samples, as shown in **Tables 1**, **2**.

**Table 1:** Univariate analysis of respiratory co-infections in hospitalized patients with acute COVID-19.

Characteristic	Bacterium or virus (n = 12)	Mycoplasma pneumoniae (n = 45)	Multi types of pathogens (n = 26)	No infection (n = 69)	Total (n = 152)	*p-*value
Sex						
Men	4	25	21	33	83	0.014
Women	8	20	5	36	69	
Age	67.0 (44.8,72.0)	59.0(47.0,73.5)	71.0 (63.5,80.5)	65.0 (46.5,75.0)	66.5 (51.5,75.0)	0.098
Family location						
East	3	5	7	9	24	0.34
West	1	2	3	4	10	
South	2	5	0	10	17	
North	2	2	3	5	12	
Centre	6	31	11	41	89	
Signs						
Fever	2	17	2	23	44	0.02
Cough	0	1	0	0	1	
Fever and cough	8	19	22	31	80	
None	2	8	2	15	27	
Lung CT change						
Single	0	4	0	7	11	0.027
Double	8	23	22	31	84	
None	4	18	4	31	57	
Hospitalized days	16.0 (7.5, 35.0)	12.0(7.5,21.0)	14.5(8.5,35.5)	9.0 (4.5,14.0)	11.0 (7.0,20.0)	0.004
Days from admission to positive SARS-CoV-2 sample	4.0(1.0,13.3)	5.0(1.0,9.5)	6.0 (1.8,20.3)	1.0 (1.0,7.0)	4.0 (1.0,9.0)	0.008
Distribution of variants						
JN.1.18.2	7	25	12	32	76	0.923
XDV.1	3	8	7	21	39	
JN.1.16	2	11	7	15	35	
KP.2	0	1	0	1	2	
Hypertension	6	21	13	22	62	0.239
Diabetes	3	13	8	13	37	0.540
Cerebral fraction	4	8	7	9	28	0.196
Coronary heart disease	3	12	5	13	33	0.760
Number of commodities						
0	4	15	7	35	61	0.49
1	4	12	7	16	39	
2	1	6	8	7	22	
3	1	11	4	9	25	
4	2	1	0	2	5	
Inpatient ward						
Internal	2	38	18	42	100	0.001
Surgery	0	0	1	5	6	
ICU	8	3	7	8	26	
Fever clinic	2	4	0	14	20	
Outcomes						
Mild	7	42	15	62	126	<0.001
Severe	5	3	11	7	26	
Ct value						
ORF1a	18.8(13.6,28.1)	19.6(15.4,22.2)	21.8(15.1,27.5)	18.9 (14.0,22.8)	19.6 (15.1,23.2)	0.156
N	18.5 (13.0,27.7)	19.2(15.6,22.7)	22.3(15.3,25.6)	18.1 (13.6,21.0)	18.9 (14.5,23.0)	0.164
Laboratory detection results						
WBC count (10^9^/L)	7.4 (5.5,10.6)	5.6 (4.3,8.1)	8.3 (6.4,13.1)	6.6 (4.9,8.2)	6.7(4.9,9.0)	0.011
Lymphocyte count (10^9^/L)	0.8 (0.4,1.3)	1.0(0.7,1.5)	0.9 (0.6,1.4)	0.9 (0.6,1.4)	0.9(0.6,1.4)	0.62
PLT (10^9^/L)	153.0(78.0,231.0)	182.0(146.0,220.0)	142.0(81.0,202.0)	195.0(135.0,276.0)	180.0 (128.0,238.0)	0.08
C-reaction protein (mg/L)	15.0(3.4,74.1)	12.4(4.3,38.0)	94.2(7.0,146.0)	13.7(3.3,41.0)	15.0(4.0,44.0)	0.14
PCT (ng/mL)	0.4 (0.1,0.9)	0.05(0.05,0.2)	1.0(0.1,3.0)	0.1(0.05,0.4)	0.1(0.05,0.5)	0.003
D-dimer (pg/mL)	4.2 (1.5,7.5)	0.9(0.2,2.7)	3.0(1.5,4.0)	1.4 (0.6,3.6)	1.6 (1.0,4.0)	0.005
ALT (IU/ML)	22.0 (11.0,69.0)	19.0(12.0,50.0)	30.0 (18.0,51.0)	22.0 (13.0,38.0)	21.0(12.0,50.0)	0.64
Lactate dehydrogenase (IU/mL)	325.0 (206.0,633.0)	194.0(167.0,236.0)	27 3.0(198.0,379.0)	220.0 (184.0,254.0)	221.0 (182.0,256.0)	0.05
IL-6 (mg/L)	19.8 (9.8,103)	6.1 (2.5,68.9)	219(2.4,709.0)	38.4 (6.5,190)	22.0(6.0,168.0)	0.63

Continuous variables presented are count or median (interquartile range). COVID-19, coronavirus disease; CT, computed tomography; SARS-CoV-2, severe acute respiratory syndrome coronavirus 2.

**Table 2:** Univariate analysis results for outcomes of patients with acute COVID-19 infection.

Characteristics	Treatment success (n = 133)	Treatment failure (n = 19)	Total (n = 152)	*p-*value
Sex				
Men	69	14	83	0.074
Women	64	5	69	
Age	65.0 (49.5,75.0)	69.0(62.03,80.0)	66.5 (51.5,75.0)	0.11
Family location				
East	23	1	24	0.04
West	6	4	10	
South	15	2	17	
North	9	3	12	
Centre	80	9	89	
Signs and symptoms				
Fever	43	1	44	0.04
Cough	1	0	1	
Fever and cough	65	15	80	
None	24	3	27	
Lung CT change				
Single	11	0	11	0.03
Double	68	16	84	
None	54	3	57	
Hospitalized days	10.0 (6.0,17.5)	20.0(10.0,34.0)	11.0 (7.0,20.0)	0.007
Days from admission to positive SARS-CoV-2 sample	3.0(1.0,9.0)	7.0(2.0,17.0)	4.0 (1.0,9.0)	0.03
Variant types				
JN.1.18.2	65	11	76	0.96
XDV.1	34	5	39	
JN.1.16	32	3	35	
KP.2	2	0	2	
Co-infection types				
No infection	65	4	69	<0.001
Mycoplasma	42	3	45	
Bacterium or virus	10	2	12	
Multi pathogens	16	10	26	
Underlying disease				
Hypertension	52	10	62	0.32
Diabetes	29	8	37	0.08
Cerebral fraction	20	8	28	0.009
Coronary heart disease	27	6	33	0.37
Number of commodities				
0	57	4	61	0.03
1	34	5	39	
2	18	4	22	
3	22	3	25	
4	2	3	5	
Hospitalized wards				
Internal	92	8	100	0.001
Surgery	5	1	6	
ICU	16	10	26	
Fever clinic	20	0	20	
Severity classification				
Mild	121	5	126	0.001
Severe	12	14	26	
ORF1a	19.3(15.1,22.8)	22.2(14.8,25.4)	19.6 (15.1,23.2)	0.33
N	18.6 (14.4,22.6)	20.5(15.1,23.3)	18.9 (14.5,23.0)	0.52
WBC count (10^9^/L)	7.2 (5.8,8.7)	6.8 (4.8,9.1)	6.8(4.9,9.3)	0.01
Lymphocyte count (10^9^/L)	0.9 (0.7,1.3)	1.0(0.3,1.8)	0.9(0.6,1.4)	0.006
PLT (10^9^/L)	207.0(117.0,248.0)	224(82.0,335.0)	183.0 (130.0,249.0)	0.007
C-reaction protein (mg/L)	18.4(8.9, 52.9)	47.7(13.4,170.8)	17.7(4.8,51.1)	< 0.001
PCT (ng/mL)	0.3 (0.1,1.2)	0.5(0.2,0.9)	0.1(0.05,0.7)	<0.001
D-dimer (pg/mL)	2.4 (0.6,6.9)	2.1(1.8,2.5)	1.6 (0.6,4.0)	<0.001
ALT (IU/mL)	16.0(12.0,28.0)	35.5(15.0,82.3)	21.8(12.9,51.1)	0.11
Lactate dehydrogenase (IU/mL)	226.0 (196.0,322.0)	252.0(225.0,307.0)	223.0 (182.0,256.0)	<0.001
IL-6(mg/L)	41.2 (11.4,173)	46.6 (9.4,904.7)	20.9 (6.0,129.0)	0.55

Continuous variables presented are count or median (interquartile range). ALT, alanine aminotransferase; ICU, Intensive Care Unit; IL-6, interleukin; PLT, platelet; PCT, procalcitonin test; WBC, white blood cell.

Continuous variables presented are count or median (interquartile range). COVID-19, coronavirus disease; SARS-CoV-2, severe acute respiratory syndrome coronavirus 2.

The Ethics Committee of the Henan Provincial People’s Hospital approved this study (Approval No. 241225). Written informed consent was obtained from all participants. All procedures adhered to the guidelines of the Declaration of Helsinki.

### 2.3 Identification and validation of SARS-CoV-2 variants

Nested polymerase chain reaction (PCR) was used to identify SARS-CoV-2 variants and their primers. The annealing temperature, amplified fragment length, and variant identification criteria are presented in **Supplementary Tables 1, 2**. High-throughput sequencing technology, a metagenomic next-generation sequencing (NGS) from Zhengzhou Autodiag Company (Zhengzhou, China) and Shanghai Sangon Biotech (Shanghai, China), was used to validate the identification of SARS-CoV-2 variants. UDG (Uracil- DNA Glycosylase) enzyme was added to the PCR reaction mixture to prevent contamination during the amplification process, Flu A, RSV, and *mycobacterium tuberculosis* had been validated the specificity, more validation details were shown in our previous study (Yuan et al., 2025).

### 2.4 Phylogenetic construction and analysis of SARS-CoV-2 variants

Among the 172 throat swabs collected, whole-genome sequencing was performed for 5 SARS-CoV-2 variants through NGS technology at Antu Company. Additionally, the whole-genome sequence (WGS) information for 23 strains belonging to Alpha, Beta, Delta, Gamma, and Omicron variant strains was downloaded from GASAID, Genebase, or the National Center for Biotechnology Information websites (GISAID, 2025; GenBase, 2024; National Library of Medicine, 2025) and aligned using SnapGene (version 6.02; GSL Biotech, Chicago, IL, USA). Furthermore, molecular evolutionary genetics analysis (MEGA) software (version 12.0.8; MEGA, USA, https://www.megasoftware.net/) was employed to construct an evolutionary tree using the best-fit substitution model of evolution and maximum likelihood method, incorporating variants currently prevalent in China. More details including accession number, nations, year, variants classification and strain name regarding WGS are summarized in **Supplementary Table 3**.

### 2.5 Clinical characteristics of hospitalized patients with COVID-19 mixed infection

The clinical data of 132 hospitalized patients and 20 outpatients treated in a fever clinic with confirmed SARS-CoV-2 infection, including seven patients co-infected with different variants, were retrieved from the hospital’s electronic medical record system (Hospital Information System). We collected variables including age, sex, city of residence in Henan Province, duration from admission to time of positive SARS-CoV-2 detection, underlying comorbidities, symptoms, cycle threshold (Ct) values for *ORF1a* and *N* genes, hospitalization duration, computed tomography (CT) features, treatment outcome, and the first laboratory detection data, which corresponded to the most recent date of positive SARS-CoV-2 infection. These data were analyzed to characterize the clinical differences between patients with respiratory mixed infection pathogens and those with a single SARS-CoV-2 infection. Mixed infection refers to both co-infection with multiple SARS-CoV-2 variants and co-infection with other non-SARS-CoV-2 respiratory pathogens.

### 2.6 Definition of treatment outcomes and severity classification

In traditional Chinese culture, the patient’s family members do not want the patient to die in the hospital and hope that the patient dies at home. The family members, especially those in rural areas, usually take the patient home prior to death; thus, the patient’s death record is unclear in the medical records. Therefore, we defined the outcomes described in the patient’s medical record prior to death: treatment failure was defined as voluntary discharge and discontinuation of treatment, whereas treatment success was defined as treatment response leading to discharge. Additionally, we classified the patients as mild and severe according to the latest Chinese guidelines of COVID-19 diagnostic and treatment criteria (Jin et al., 2020).

### 2.7 Statistical analysis

For univariate analyses, summary statistics are presented as medians with interquartile ranges (IQRs) or means with standard errors, depending on the distribution. Statistical significance was assessed using the Kruskal–Wallis one-way analysis of variance test with Bonferroni adjustments for continuous variables. Categorical variables were analyzed using Pearson’s χ² or Fisher’s exact tests. Variables with *p* values <0.1 in the univariate analyses were included in the multivariate logistic regression analysis. Backward stepwise logistic regression was used to evaluate the risk factors for COVID-19 in patients with mixed respiratory infections and treatment failure. Eighty-day survival curves were constructed using the Kaplan–Meier method. Event was defined as treatment outcome of patient including treatment success or treatment failure during 80 days of hospitalization. Moreover, censoring defined situations where, at the end of the study or during follow-up, a patient has not experienced the event we defined, or for other reasons, we can no longer obtain complete follow-up information for that patient. Log-rank tests were performed using Prism (version 8.0; GraphPad, La Jolla, CA, USA) software. The significance threshold for group differences was set at *p* < 0.05. All analyses were performed using SPSS (version 25.0; IBM Corp., Armonk, NY, USA) and GraphPad Prism.

## 3 Results

### 3.1 Characteristics of patients co-infected with different SARS-CoV-2 variants

**Figure 1:**
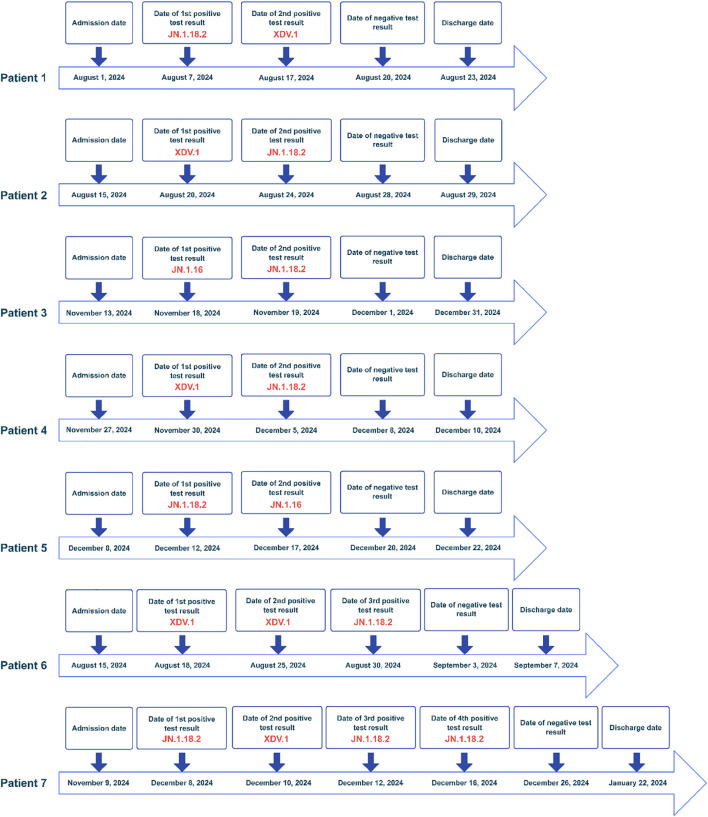
Timeframe of seven patients with acute COVID-19 infected with different SARS-CoV-2 variants (red text). The SARS-CoV-2 variant of the first sample of patient 6 was validated by WGS. COVID-19, coronavirus disease; WGS, whole-genome sequence; SARS-CoV-2, severe acute respiratory syndrome coronavirus.

**Figure 2:**
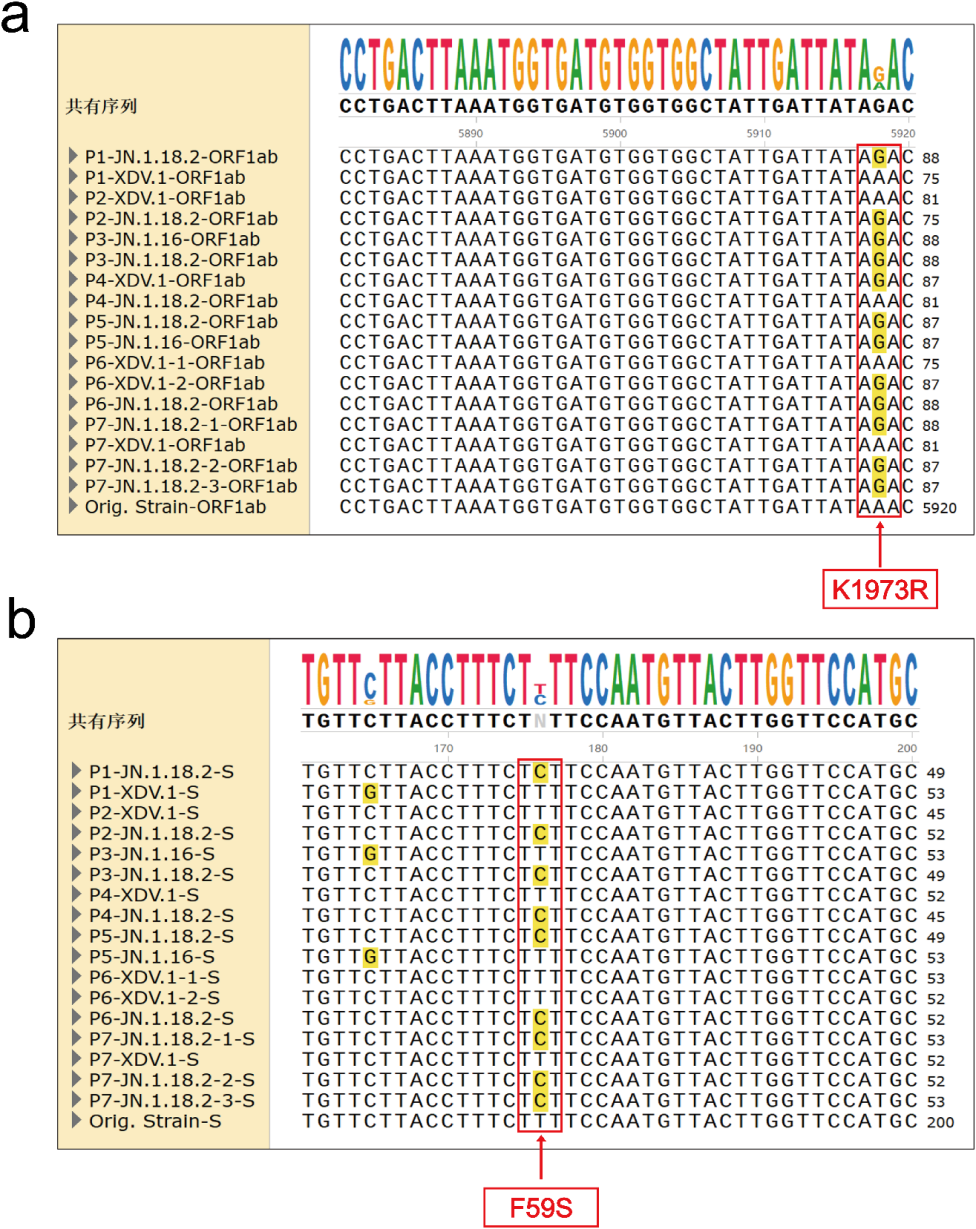
Comparison of mutation points in nucleotide base sequences among different variants from the same host. These comparisons were done on different dates for seven patients. **(A)** Different F59S mutations of the spike gene in nucleotide base sequences between XDV.1, JN.1.18.2 and JN.1.16 variants from seven patients. **(B)** Different K1973R mutation of the *ORF1a* gene in nucleotide base sequences between XDV.1, JN.1.18.2 and JN.1.16 variants from seven patients.

**Figure 3:**
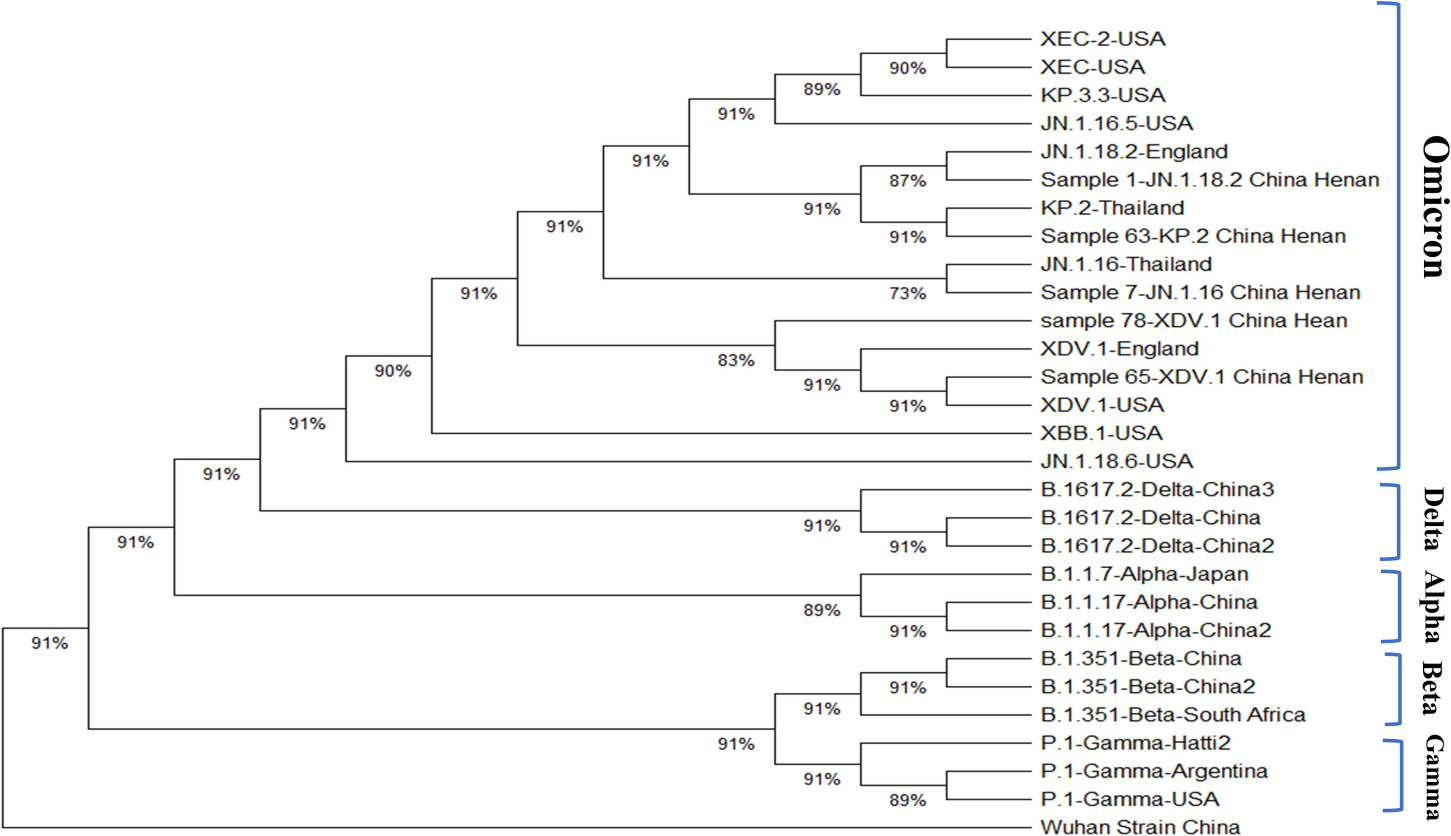
Phylogenetic tree based on the WGS data of different SARS-CoV-2 variants. The sample 78 is the first positive sample from patient 6 in **Figure 1**. SARS-CoV-2, severe acute respiratory syndrome coronavirus 2; USA, United States of America; WGS, whole-genome sequence.

When identifying the new coronavirus variants using nested PCR, we identified cases of mixed infection with different variants. Among 172 throat swab samples from 152 patients, 15 had at least two consecutive samples collected. Among these 15 patients, 7 had different SARS-CoV-2 variants detected by nested PCR and NGS in samples collected on different dates from the same patient (**Table 3**, **Figure 1**, **2**). The detection rate of mixed infection with different SARS-CoV-2 variants was 46.7% (7/15) among all patients with more than two samples. Among the seven patients infected with different variants, five had two positive samples, one had three positive samples, and one patient had four positive samples. We designated patients as patient 1 through 7 (**Figure 1**). Among them, patient 1 was a 71-year-old male who died after staying in the intensive care unit (ICU) for 22 days due to a mixed infection of respiratory Flu B, *Acinetobacter baumannii*, and *Candida albicans.* He had a cerebral infarction and was diagnosed with COVID-19 after spinal surgery on 1 August 2024. A positive SARS-CoV-2 throat swab sample was documented on the 7^th^ and 17^th^ of August 2024. However, these two positive samples were identified as the JN.1.18.2 and XDV.1 variants using nested PCR and NGS, respectively. To treat acute COVID-19, oral antiviral drugs oseltamivir and monoravir were administered for 10 d. However, the patient died due to severe comorbidities. Therefore, the mortality rate was 14.3% (1/7) among seven patients co-infected with different SARS-CoV-2 variants, which was higher than the average mortality rate of all patients with COVID-19 (3.3%, 5/152; *p* < 0.001). The other six patients had at least two positive SARS-CoV-2 samples, each containing different variants identified as JN.1.16, JN.1.18.2, or XDV.1, within the same host on different dates (**Figures 1**, **2**). As shown by the phylogenetic tree constructed for the WGS results of five variant strains and downloaded sequences of other 23 SARS-CoV-2 variants, the five selective and representative variant strains from Henan Province and same Omicron variants from Thailand, the United States, and England, were clustered together (**Figure 3**). These are the Omicron variants currently circulating in the world. Furthermore, the phylogenetic tree showed that the XDV.1 variant was closer to the Wuhan prototype, whereas JN.1.16 and JN.1.18.2 had a longer evolutionary distance (**Figure 3**). Overall, patients with mixed COVID-19 infections of different variants had distinct clinical characteristics compared to those without; they were older [73(66,85)] and had more underlying diseases, such as hypertension, diabetes, chronic heart disease, and cerebral infarction. Moreover, in relation to other patients, these patients had a higher mixed infection rate with other respiratory pathogens (85.5% vs 54.6%) and higher severe COVID-19 rate in accordance with previous guidelines (42.8% [3/7] vs. 17.1% [26/152]; *p* < 0.001] (Jin et al., 2020)). Common respiratory pathogens in patients with acute COVID-19 included *M. pneumoniae, Klebsiella pneumoniae*, and *Aspergillus.* Additionally, 71.4% (5/7) of patients with mixed infection variants were treated with antiviral drugs such as nirmatrelvir, ritonavir, or molnupiravir (**Table 3**).

**Table 3:** Demographics of seven patients with acute COVID-19 infected with different SARS-CoV-2 variants.

Case No.	Sex	Age (years)	Underlying disease	Number of days hospitalized	Geolocation in henan province	No. of positive SARS-CoV-2 samples	Outcome	Respiratory pathogens of mixed infection	Admission diagnosis	Inpatient ward	Anti-virus drug usage
1	Man	71	Cerebral infraction	22	Center	2	Death	Flu B, Acinetobacter baumannii, Candida albicans	After the spinal surgery	Comprehensive ICU	Oseltamivir, Monoravir
2	Man	73	Hypertension, diabetes, CHD	9	South	2	Remission	Aspergillus, Nocardia, *Staphylococcus aureus*	Lymphatic multiplication	Infectious disease	Monoravir
3	Man	85	Cerebral infraction, diabetes	47	West	2	Remission	Aspergillus, Candida Tropicalis. Human herpesvirus	Pneumonia	Respiratory ICU	No
4	Man	66	None	13	Center	2	Remission	None	Amyotrophy	Respiratory ICU	Nematavir, Ritonavir
5	Man	61	Hypertension, Cerebral infarction, CHD	14	Center	2	Remission	Mycoplasma pneumoniae	Cerebrovascular disease	Geriatrics	Nematavir, Ritonavir
6	Man	88	Hypertension, CHD	73	Center	4	Remission	Klebsiella pneumoniae	Fever	Geriatrics	Monoravir
7	Man	82	Hypertension, Cerebral infraction, CHD	12	Center	3	Remission	Mycoplasma pneumoniae	Fever	Geriatrics	No

CHD, coronary heart disease; COVID-19, coronavirus disease; ICU, Intensive Care Unit; SARS-CoV-2, severe acute respiratory syndrome coronavirus 2.

### 3.2 Clinical characteristics of patients co-infected with respiratory pathogens

The clinical characteristics of patients co-infected with other respiratory pathogens and those without such mixed infections were further investigated in the univariate analysis (**Table 1**). Among the 152 patients with COVID-19, 83 had co-existing respiratory pathogen infections, resulting in a total respiratory pathogen mixed infection rate of 54.6%. The most common co-existing respiratory pathogen was *M. pneumoniae*, with a mixed infection rate of 29.6% (45/152), followed by the group with mixed infections of multiple pathogens consisting of at least two or more types of mycoplasmas, bacteria and viruses, and the group infected with either bacteria or viruses, with an infection rate of 17.1% (26/152) and 7.9% (12/152), respectively. Significant differences in the mixed infection rates were observed among patients infected with different pathogens (*p* < 0.001).

**Figure 4:**
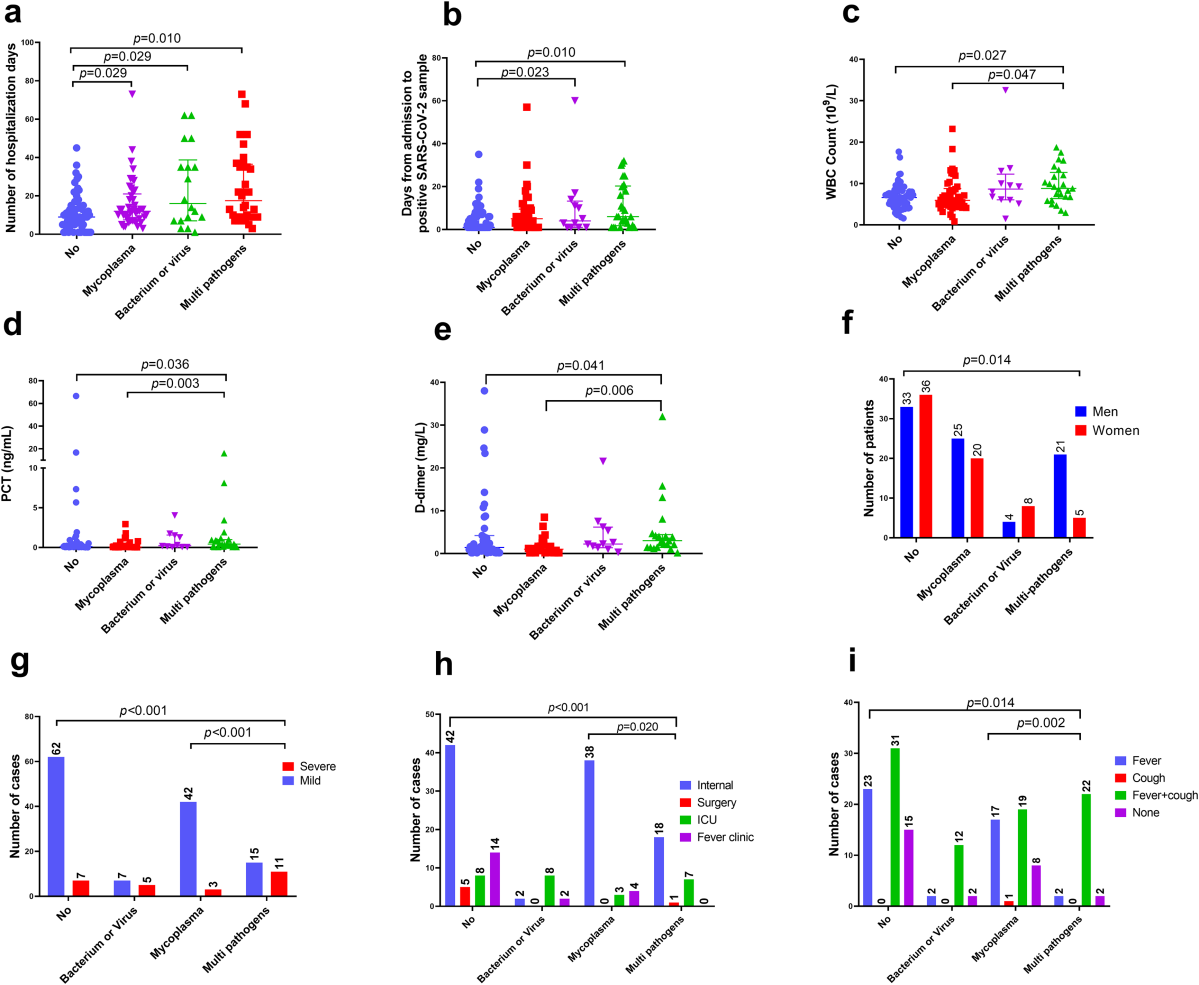
Comparison of clinical characteristics among patients with acute COVID-19 co-infected with different pathogens. **(A)** Hospitalization days, **(B)** days from admission to positive SARS-CoV-2 sample detection, **(C)** white blood cell (WBC) counts, **(D)** platelet count (PCT) concentration, **(E)** D-dimer concentration, **(F)** sex, **(G)** numbers of severe and mild cases, **(H)** number of cases in the hospital ward, and **(I)** number of patients with signs and symptoms. SARS-CoV-2, severe acute respiratory syndrome coronavirus 2; COVID-19, coronavirus disease; ICU, intensive care unit.

**Figure 5:**
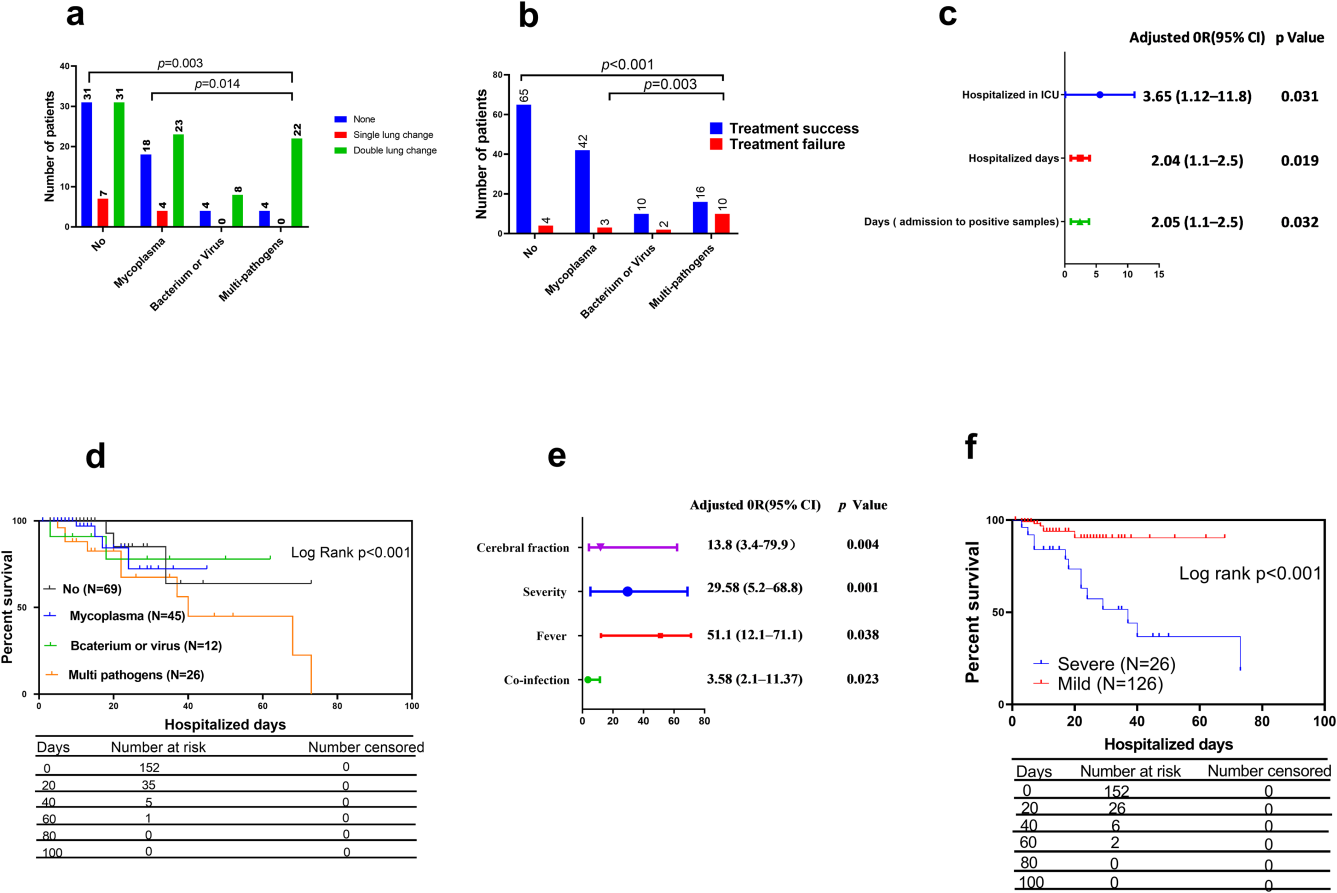
Multivariate and survival analysis results for patients with acute COVID-19 co-infected with different pathogens. **(A)** Number of patients with lung computed tomography (CT) change, **(B)** number of patients with different treatment outcomes, **(C)** risk factors for patients with mixed infection, **(D)** survival analysis for different pathogens, **(E)** risk factors for patients with treatment failure, and **(F)** survival analysis for different severity types. CI, confidence interval; COVID-19, coronavirus disease; OR, odds ratio.

Infections involving multiple pathogens had a median hospitalization time of 14.5 d (IQR: 8.5–35.5 d), which was significantly longer than that of those without co-existing respiratory infection (median: 9.0 d, IQR: 4.5–14.0 d; *p* = 0.010, **Figure 4A**). Patients with mixed infections involving multiple pathogens (*p* = 0.010) and bacterium or virus (*p* = 0.023) had a longer duration from admission to positive SARS-CoV-2 sample detection than those without co-existing respiratory infections (**Figure 4B**). Additionally, patients infected with multiple pathogens had more elevated white blood cell (WBC) counts, procalcitonin (PCT), and D-dimer levels than those infected with *M. pneumonia* and without mixed infection (**Figures 4C–E**). Patients with mixed infections involving multiple pathogens were more likely to be male (**Figure 4F**); have a severe classification (**Figure 4G**); be hospitalized in the ICU (**Figure 4H**); have fever and cough (**Figure 4I**), double lung changes (**Figure 5A**), and treatment failure (**Figure 5B**) than those infected with *M. pneumonia* or without mixed infection. Multivariate analysis confirmed that ICU hospitalization (odds ratio [OR] = 3.65 (1.25-5.03), *p* = 0.031), number of hospitalization days (OR = 2.04 (1.2-3.02), *p* = 0.019), and days from admission to positive SARS-CoV-2 sample detection (OR = 2.05 (1.32-3.86), *p* = 0.032) were independent risk factors for acute COVID-19 mixed with different respiratory pathogens (**Figure 5C**). Survival analysis also indicated that patients with mixed infections involving multiple pathogens had a lower survival rate compared to those without mixed infection (log rank *p* < 0.001, **Figure 5D**).

No significant differences in age, SARS-CoV-2 variant distribution, geographic location, or underlying comorbidities were observed between patients infected with and without different pathogens. Similarly, no significant differences in laboratory detection results were found between patients with and without different pathogen mixed infections (**Table 1**).

### 3.3 Outcomes of patients mixed infected with respiratory pathogens

To identify the clinical characteristics of patients with COVID-19 co-infected with different respiratory pathogen***s***, we compared the characteristics of patients regarding treatment success and failure. Of 152 patients with COVID-19, 26 had severe infections according to the latest Chinese guidelines for COVID-19 treatment and diagnostic criteria (Jin et al., 2020), 19 had a record of treatment failure, reflecting a total treatment failure rate of 12.5% (**Table 2**).

Patients with treatment failure had a median hospitalization time of 45 d (IQR: 20.5–71.8 d), significantly longer than that of those with treatment success (median: 11 d, IQR: 5.5–24.5; *p* = 0.007; **Supplementary Figure 1A**). Patients with treatment failure had a longer duration from admission to positive SARS-CoV-2 sample detection than those with treatment success (*p* = 0.028; **Supplementary Figure 1B**). Additionally, patients with treatment failure had more elevated WBC counts (**Supplementary Figure 1C**), PCT (**Supplementary Figure 1D**), CRP (**Supplementary Figure 1E**), D-dimer (**Additional File 4** (**F)**), and lactate dehydrogenase (**Supplementary Figure 1H**) than those with treatment success. Conversely, patients with treatment failure had fewer lymphocyte (*p* = 0.006; **Supplementary Figure 1G**) and platelet counts than those with treatment success (*p* = 0.007; **Supplementary Figure 2A**). Patients with treatment failure were mostly from the Eastern region of Henan Province, China (**Supplementary Figure 2B**). They had a greater incidence of fever and cough (**Supplementary Figure 2C**), double lung CT change (**Supplementary Figure 2D**), and cerebral fraction (**Supplementary Figure 2E**). Correspondingly, patients with treatment failure also had a greater number of underlying diseases than those with treatment success (**Supplementary Figure 2F**), with ICU hospitalizations (**Supplementary Figure 2G**) and more frequent severity classifications (**Supplementary Figure 2H**). Furthermore, multivariate analysis showed that mixed infection with multiple pathogens (OR = 3.58(1.34-6.67), *p* = 0.023), severity classification (OR = 29.58(8.65-44.1), *p* = 0.001), cerebral fraction (OR = 51.1(2.5-66.7), *p* = 0.038), and fever (OR = 13.8(2.23-25.6), *p* = 0.004) were independent risk factors for acute COVID-19 infection with treatment failure (**Figure 5E**). Survival analysis also indicated that patients with a severity classification had a lower survival rate than those with mild classifications (log rank *p* < 0.001, **Figure 5F**).

No significant differences in age, sex, SARS-CoV-2 variant distribution, and underlying comorbidities were observed between patients with treatment failure and success. No significant differences in laboratory parameters were noted between patients with treatment failure and success (**Table 2**).

## 4 Discussion

Simultaneous mixed infection with different viruses can negatively impact disease progression (Sayama et al., 2024). However, the symptomatology of mixed infection with different lineages of the same virus remains unknown. In this study, three of the seven patients developed severe symptoms, including fever and cough, and were diagnosed with respiratory infections. They were transferred to the ICU, and one died due to other comorbidities. To the best of our knowledge, this is the first report of mixed infection with different SARS-CoV-2 variants in patients in China. Though the samples for detection of different SARS-CoV-2 variants were collected on different dates from the same patient, it does not necessarily indicate true co-infection (Rockett et al., 2022; Castillo et al., 2024). This may also reflect sequential infection, superinfection, or variant replacement over time (Santiago et al., 2023).

Unlike other reports where mixed infection with different variants was detected in the same sample (Sayama et al., 2024; Somekh et al., 2021), in our study, different variants were detected in samples collected on different dates but in the same host; this appears to be a new mixed infection characteristic that has not been reported elsewhere. This may be because the patient was first infected with a SARS-CoV-2 variant after being admitted to the hospital. Since the patient’s condition was relatively severe and strict quarantine measures, including wearing a mask and maintaining social distancing, were not enforced, the patient was subsequently infected with another type of SARS-CoV-2 variant following contact with other patients infected with SARS-CoV-2 variants. Mixed infection with different SARS-CoV-2 variants accounted for 46.7% (7/15) of all cases with more than two samples, indicating that mixed infection with different variants in patients with COVID-19 has been underestimated in the past and that the detection of SARS-CoV-2 variants in inpatients, especially in older adult patients with comorbidities, should be strengthened in the future. During hospitalization, control measures for nosocomial infections in patients with COVID-19 should be improved to protect them from a second SARS-CoV-2 infection (Livieratos et al., 2024). Another reason for this finding may be that the virus mutated after antiviral drug administration in these patients (Sasaki et al., 2024). However, we believe this is unlikely because the interval between detecting two different variants in patients was very short, approximately 3–10 days. Additionally, the emergence of SARS-CoV-2 variants is generally attributed to the virus’s active evolution to evade human immune responses. There are similar reports on genetic recombination of different variants in patients with HIV, resulting in new variants (Mandala and Liu, 2021; Song et al., 2024).

More than half of the patients with COVID-19 had mixed infections with other respiratory pathogens, such as *M. pneumoniae, K. pneumoniae, and Aspergillus*. These patients had longer hospital stays, longer detection intervals from admission to positive SARS-CoV-2 samples, and a greater chance of developing severe disease and treatment failure. Therefore, in clinical practice, early detection of WBC count, PCT, and D-dimer concentration can indeed help predict severe cases and treatment outcomes caused by mixed infections (Zhao et al., 2022).

In the seven cases of mixed infection with different SARS-CoV-2 variants, three variants were common: JN.1.18.2, JN.1.16, and XDV.1. As demonstrated in the WGS evolutionary tree, the JN.1.18.2 and JN.1.16 series variants were grouped into one category. However, XDV.1 and the Wuhan prototype were closer in evolution, indicating that the JN.1.18.2 variant had a longer evolutionary distance than that of the XDV.1 variant. Among the currently prevalent variants in the Henan region, the JN.18.1.2 variant accounted for the vast majority, which is consistent with the research result of Zhang et al., which also identified JN.18.1.2 as the dominant variant in China (Zhang et al., 2023). Considering the presence of a new spike protein mutation (F59S) in JN.1.18.2 compared to that of XDV.1, this variant may have a stronger immune escape capability. This aligns with global trends reported in the literature, where JN.1-related variants, including KP.2 and XEC, are among the most prevalent worldwide (Li et al., 2024; Lewnard et al., 2024; Suthar et al., 2025).

Compared with other literature on the risk factors for mixed infection with other pathogens and treatment failure in patients with COVID-19 (Gan et al., 2024; Piechowicz et al., 2024), our unique factors include a longer hospital stay, the time from hospitalization to the detection of positive COVID-19 specimens, and the presence of underlying cerebral infarction in the patients. These unique characteristics suggest that clinicians consider possible nosocomial infection control in these patients to prevent re-infection of the SARS-CoV-2 variant and other respiratory pathogens and reduce treatment failure rate of severe patients (Carpenter et al., 2023; Andrés et al., 2022; Blot et al., 2022).

This study has some limitations. First, we only had a small number of samples because of the few positive SARS-CoV-2 samples per month in 2024. More samples are required for collection in 2025 to validate the clinical characteristics of patients with mixed infection variants. Second, some samples with mixed infection variants identified by nested PCR had lower viral loads, and WGS was not conducted to verify the correction of identification. Third, nested PCR requires two rounds of amplification, and the results need to be viewed by agarose gel electrophoresis, which is a bit cumbersome in operation. The next step is to improve and optimize it. We plan to explore using multi-primed probes for real-time PCR to identify SARS-CoV-2 variants. Fourth, we do not have a level 3 biology-safe lab; thus, we cannot culture live SARS-CoV-2 positive samples to obtain high-quality and complete viral RNA for further study.

### 4.1 Conclusions

We reported mixed infections with different SARS-CoV-2 variants in seven patients with acute COVID-19, identified from various samples and dates within the same host. These patients were older and had more underlying conditions. Due to longer hospitalization, they were more susceptible to developing severe cases of infection and were co-infected with another variant and additional pathogens. Early detection of laboratory parameters, including WBC count, PCT, and D-dimer concentrations, can help clinicians diagnose and treat patients with severe acute COVID-19 to reduce mortality. In the context of the SARS-CoV-2 pandemic, active variant surveillance and analysis of clinical data are essential to understand the relationship between mixed infection and disease progression (Alemán et al., 2025).

## Data Availability

The original contributions presented in the study are included in the article/**Supplementary Material**. Further inquiries can be directed to the corresponding authors.
