# Supplementary material for: Distinct characteristics on mixed infection of SARS-CoV-2 variants and other respiratory pathogens among patients with acute COVID-19 in central China
Source: Front Cell Infect Microbiol. 2026 Mar 18;16:1653022. doi: 10.3389/fcimb.2026.1653022 (PMC13038880; doi:10.3389/fcimb.2026.1653022)
Supplement: Supplementary file 1 [file Table1.docx]

Supplementary Material

# Supplementary Figures and Tables

## Supplementary Figures


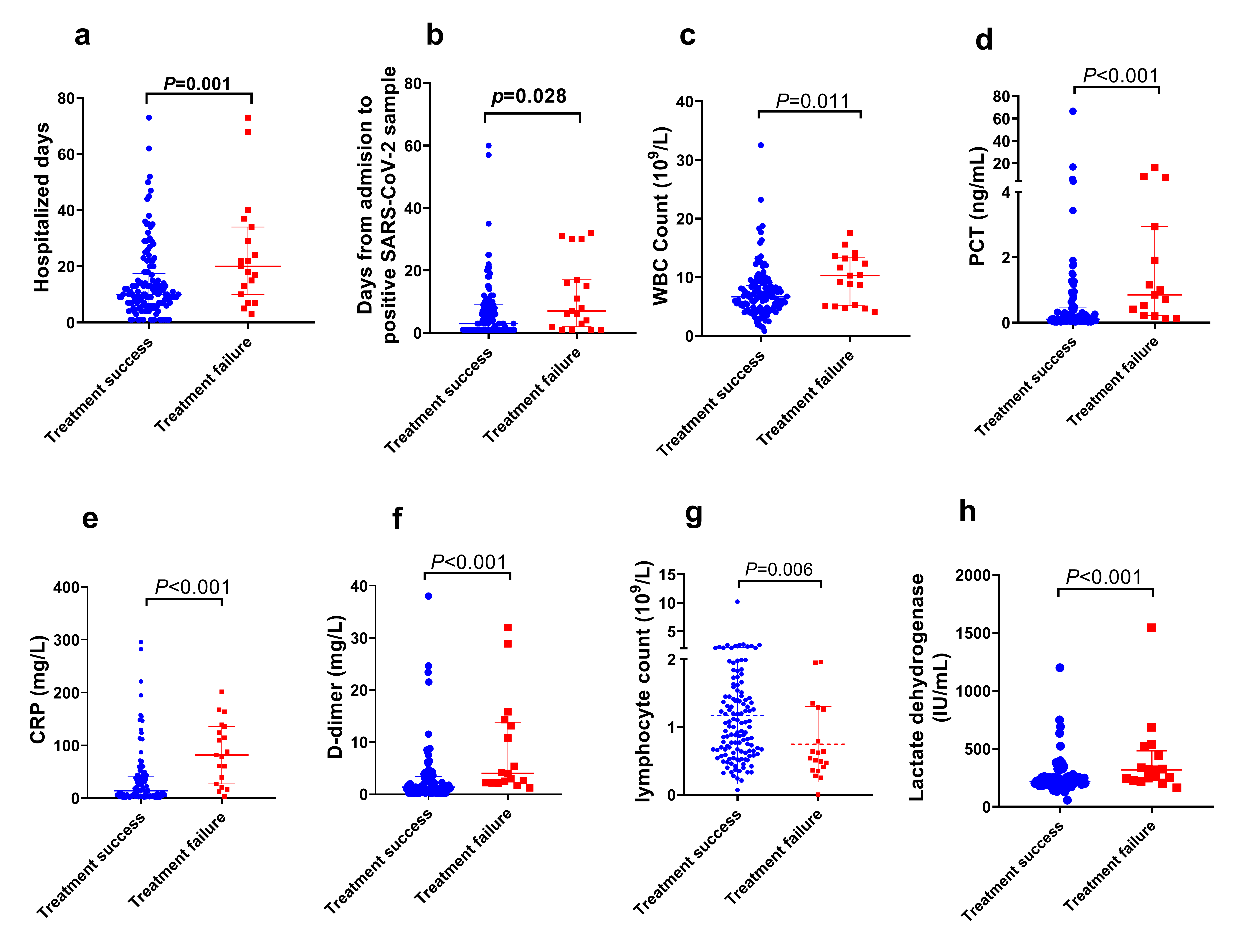


**Supplementary Figure 1.** Comparative analysis of clinical outcomes between treatment failure and success groups in patients with acute COVID-19. (**A**) Duration of hospitalisation, (**B**) Time from admission to SARS-CoV-2 nucleic acid positivity, (**C**) WBC counts, (**D**) Procalcitonin (PCT) concentration dynamics, (**E)** C-reactive protein (CRP) concentration, (**F**) D-dimer elevation patterns, (**G)** Lymphocyte counts, (**H**) Lactate dehydrogenase concentration. SARS-CoV-2, severe acute respiratory syndrome coronavirus 2; WBC, white blood cells; COVID-19, coronavirus disease.


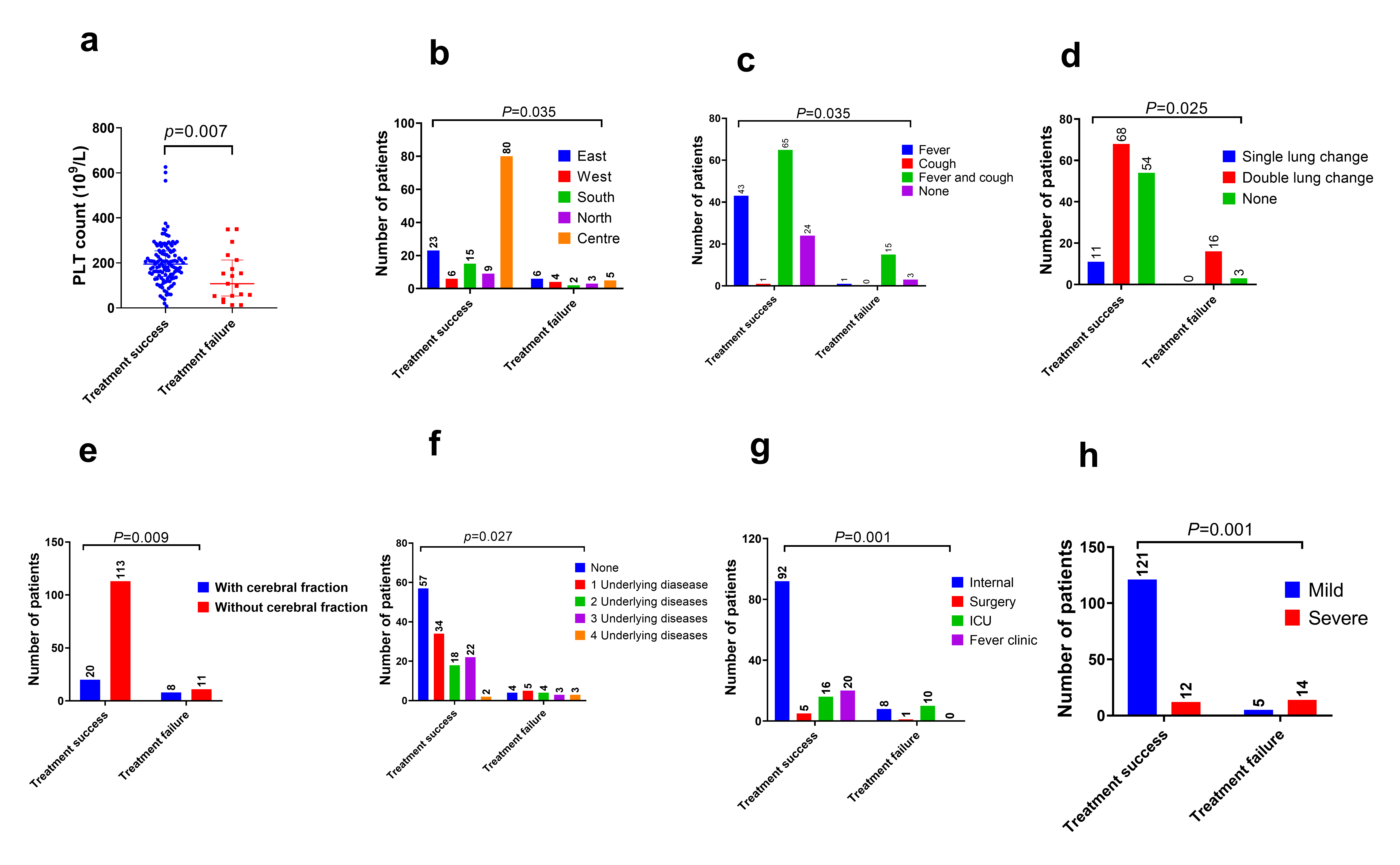


**Supplementary Figure 2.** Comparative analysis of outcome characteristics between treatment failure and success groups in patients with acute COVID-19. (**A**) Platelet counts, (**B)** Geographical distribution of patients across prefectures within Henan Province, (**C**) Frequency of presenting clinical signs and symptoms, (**D**) Radiographic alterations on thoracic CT imaging, (**E**) Incidence of cerebral infarction, (**F**) Comorbidity burden stratification, (**G**) Hospital ward allocation patterns, (**H)** Disease severity stratification (severe vs mild presentations). PLT, platelets; COVID-19, coronavirus disease; CT, computed tomography; ICU, intensive care unit.

## Supplementary Tables

**Supplementary Table 1.** Details of nested primers used for identifying variants of severe acute respiratory syndrome coronavirus 2

| **Primer name** | **Amplifying area and position** | **Direction** | | **Sequence（5′–3′）** | **Annealing temperature** | **Product**  **length** | **Specific point of variants** |
| --- | --- | --- | --- | --- | --- | --- | --- |
| O379 | ORF1a5768-6166 | F | | CAAGCTTCGATAATTTTAAGTTT | 50 | 379 | K1973R |
|  |  | R | | TAGGATTTTCCACTACTTCT |  |  |  |
| O330n | ORF1a5793 -6142 | F | | ATGTGATAATATCAAATTTGCT | 50 | 330 |  |
|  |  | R | | GACTGGTTTTAGATCTTCG |  |  |  |
| O268 | ORF1a5752-6019 | F | | CAACCATATCCAAACGCAAG | 50 | 268 | K1973R |
|  |  | R | | TATTTGGTTTATACGTGGCT |  |  |  |
| 0190n | ORF1a5794-5983 | F | | TGTGATAATATCAAATTTGCTG | 50 | 190 |  |
|  |  | R | | TAACATGCCAAACAATAGGT |  |  |  |
| O220 | ORF1a5787-5807 | F | | GTTTGTATGTGATAATATCAAA | 48 | 220 | K1973R |
|  |  | R | | TGTTAACATGCCAAACAAT |  |  |  |
| O156n | ORF1a5811-5967 | F | | TGCTGATGATTTAAACCAGT | 50 | 156 |  |
|  |  | R | | GGTTTATGTAACAATTTAGCTC |  |  |  |
| S334 | S83-416 | F | | TACACTAATTCTTTCACAC | 48 | 334 | F59S |
|  |  | R | | AAAATGGATCATTACAAA |  |  |  |
| S292n | S105-396 | F | | TGTTTATTACCCTGACAAA | 50 | 292 |  |
|  |  | R | | TGTTAGACTTCTCAGTGGAA |  |  |  |
| S229 | S81-309 | F | | TACACTAATTCTTTCACACG | 48 | 229 | F59S |
|  |  | R | | TGTTAGACTTCTCAGTGGAA |  |  |  |
| S161n | S103-263 | F | | GTGTTTATTACCCTGACAAA | 50 | 161 |  |
|  |  | R | | CATCATTAAATGGTAGGACA |  |  |  |
| S202 | S81-283 | F | | ATACACTAATTCTTTCACACG | 48 | 202 | F59S |
|  |  | R | | GGAAGCAAAATAAACACCATC |  |  |  |
| S135n | S103-238 | F | | GGTGTTTATTACCCTGACA | 50 | 135 |  |
|  |  | R | | AAACCTCTTAGTACCATTGGTC |  |  |  |
| S220 | S1331-1551 | F | | GCATAGTGGTAATTATGAT | 48 | 220 | Q493E |
|  |  | R | | TAGAAGTTCAAAAGAAAGTAC |  |  |  |
| S178n | S1352-1530 | F | | ACTGGTATAGATCGCTTAGG | 50 | 178 |  |
|  |  | R | | ACTACTCTGTATGGTTGGT |  |  |  |
| S300 | S1262-1561 | | F | ATAATTATAAATTACCAGATGA | 48 | 300 | L455S  F456L  Q493E |
|  |  |  | R | GTGCATGTAGAAGTTCAAA |  |  |  |
| S248n | S1287-1534 | | F | TACAGGCTGCGTTATAGCTT | 48 | 248 |  |
|  |  |  | R | CTACTACTCTGTATGGTTGG |  |  |  |
| S210 | S1235-1444 | | F | CAGGGCAAACTGGAAA | 50 | 210 | L455S  F456L |
|  |  |  | R | CTTTACAAGGTTTGTTACCG |  |  |  |
| S159n | S1265-1423 | | F | ATTATAAATTACCAGATGATT | 50 | 159 |  |
|  |  |  | R | CCTGATAGATTTCAGTTGA |  |  |  |
| S280 | S1276-1556 | | F | CCAGATGATTTTACAGG | 48 | 280 | L455S, F456L,  Q493E |
|  |  |  | R | GTAGAAGTTCAAAAGAAAG |  |  |  |
| S242n | S1294-1536 | | F | TGCGTTATAGCTTGGAA | 50 | 242 |  |
|  |  |  | R | ACTACTACTCTGTATGGT |  |  |  |
|  | | | | | | | |
| S234 | S1346-1579 | | F | ATGATTACTGGTATAGATCGCTT | 50 | 234 | Q493E |
|  |  |  | R | TAGGTCCACAAACAGTTGC |  |  |  |
| S190n | S1369-1558 | | F | GGAAGTCTAAACTCAAACCTT | 50 | 195 |  |
|  |  |  | R | GGTGCATGTAGAAGTTCAAA |  |  |  |
| S301 | S3218-3517 | | F | AGAACTTCACAACTGC | 48 | 301 | P1143L, V1104L |
|  |  |  | R | TAATGCCAGAGATGTCA |  |  |  |
| S265-1n | S3235-3499 | | F | CCTGCCATTTGTCATG | 48 | 265 |  |
|  |  |  | R | CTAAATCAACATCTGGT |  |  |  |
| S265 | S3242-3506 | | F | TTTGTCATGATGGAAAAGCAC | 48 | 265 | P1143L |
|  |  |  | R | ATGTCACCTAAATCAACATCTG |  |  |  |
| S200n | S3264-3463 | | F | CTTTCCTCGTGAAGGTGTC | 48 | 200 |  |
|  |  |  | R | ATTTATCTAACTCCTCCTTGAA |  |  |  |
| S260 | S928-1188 | | F | AAGGAATCTATCAAACTTC | 48 | 260 | R346T |
|  |  |  | R | ATAGACATTAGTAAAGCAGA |  |  |  |
| S216n | S949-1164 | | F | AACTTTAGAGTCCAACCAA | 48 | 216 |  |
|  |  |  | R | ATTTAATTTAGTAGGAGACAC |  |  |  |
| S250 | S933-1182 | | F | AATCTATCAAACTTCTAACTT | 50 | 250 | R346T |
|  |  |  | R | ATTAGTAAAGCAGAGATCATT |  |  |  |
| S195n | S956-1150 | | F | GAGTCCAACCAACAGAATC | 50 | 195 |  |
|  |  |  | R | GAGACACTCCATAACACTT |  |  |  |
| N343 | N536-878 | | F | GCAGTCAAGCCTCTTCTCGTT | 50 | 343 | G204R G204P Q229K |
|  |  | | R | CTGATTAGTTCCTGGTCCCCAA |  |  |  |
| N280n | N561-840 | | F | CTCATCACGTAGTCGCAACAG | 50 | 280 |  |
|  |  | | R | TGGACCACGTCTGCCGAAA |  |  |  |

**Supplementary Table 2.** Specific mutation points in the Spike protein, ORF1a, and N proteins, indicating the specific variant sites present in the S gene, N gene and O gene of different COVID-19 variants.

| **Variants** | **Spike** | | | | | | | | **ORF1a** | **N** | | | |
| --- | --- | --- | --- | --- | --- | --- | --- | --- | --- | --- | --- | --- | --- |
|  | **F59S** | **L455F** | **L455S** | **F456L** | **Q493E** | **R346T** | **V1104L** | **P1143L** | **K1973R** | **R203K** | **G204R** | **G204P** | **Q229K** |
| JN.1.18.2 | yes | no | yes | yes | no | yes | no | yes | yes | yes | yes | no | yes |
| JN.1.18 | no | no | yes | yes | no | yes | no | yes | yes | yes | yes | no | yes |
| JN.1 | no | no | yes | no | no | no | no | Yes | yes | yes | yes | no | yes |
| JN.1.16 | no | no | yes | yes | no | no | no | Yes | yes | yes | yes | no | yes |
| KP.2 | no | no | yes | yes | no | yes | yes | Yes | yes | yes | yes | no | yes |
| KP.3 | no | no | yes | yes | yes | no | yes | Yes | yes | yes | yes | no | yes |
| XDV | no | no | yes | no | no | no | no | Yes | no | yes | yes | no | no |
| XDV.1 | no | no | yes | yes | no | no | no | Yes | no | yes | yes | no | no |
| XEC | yes | no | yes | yes | yes | no | yes | Yes | yes | yes | no | yes | yes |
| EG.5.1 | no | yes | no | yes | no | no | no | No | no | yes | yes | no | no |
| HK.3 | no | yes | yes | yes | no | no | no | No | no | yes | yes | no | no |
| HK.3.2 | no | yes | yes | yes | no | no | no | No | no | yes | yes | no | no |

**Supplementary Table 3.** Accession numbers of the genomic sequences of the representative SARS-CoV-2 variants, indicating the sources of the variant sequences required for drawing the evolutionary tree diagram

| **Virant classification** | **Strain name** | **Year** | **Nation** | **Accession No.** |
| --- | --- | --- | --- | --- |
| Protype | SARS-CoV-2 Wuhan Hu-1 | 2019 | China | **NC_045512** |
| Omicron-XEC | NY-PRL-250311- | 2025 | USA | **PV274928** |
| Omicron-JN.1.18.6 | CO-CDPHE-43093558- | 2025 | USA | **PV275462** |
| Omicron-JN.1.18.2 | MONOPARTITE | 2024 | England | **OZ118877.1** |
| Omicron-KP.3 | MD-CDC-LC1134043- | 2025 | USA | **PV258064.1** |
| Omicron-XBB.1 | NY-GBW-VWPOAAAZ126980- | 2023 | USA | **PQ017092.1** |
| Omicron-KP.2 | THA/CU-AMV73- | 2024 | Thailand | **PQ549827.1** |
| Omicron-XDV.1 | USA/CA-CDPH-500139739 | 2024 | USA | **PV260708.1** |
| Omicron-JN.1.16 | THA/CU-A24113-CSV- | 2024 | Thailand | **PQ107753.1** |
| Alpha-B.1.1.7 | SB_NCGM_SARS_COV_2_00142 | 2021 | Japan | **BS007920.1** |
| Alpha-B.1.1.7 | CELL PASSAGE/CHN/CLONE 1/2021 | 2021 | China | **OQ231280.1** |
| Alpha-B.1.1.7 | CELL PASSAGE/CHN/CLONE 2/2021 | 2021 | China | **OQ231281.1** |
| Alpha-B.1.1.7 | INEI104808 | 2021 | Argentina | **OZ060934.1** |
| Beta-B.1.351 | ZAF/NHLS-UCT-GS-9232/2020 | 2020 | South Africa | **PP522432** |
| Beta-B.1.351 | MONOPARTITE | 2020 | England | **OY868446.1** |
| Beta-B.1.351 | CHN/GDIPH-GDPCC-04/2021 | 2021 | China | [**PP226969.1**](https://www.ncbi.nlm.nih.gov/nuccore/PP226970.1) |
| Beta-B.1.351 | CHN/GDIPH-GDPCC-05/2021 | 2021 | China | [**PP226970.1**](https://www.ncbi.nlm.nih.gov/nuccore/PP226970.1) |
| Delta-B.1.617.2 | /CHN/S197-2056-341191/2021 | 2021 | China | **PP380238.1** |
| Delta-B.1.617.2 | /CHN/S179-1843-327183/2021 | 2021 | China | **PP380230.1** |
| Delta-B.1.617.2 | CHN/S187-1900-334195/2021 | 2021 | China | **PP380234.1** |
| Gamma-P.1 | USA/1PD1N/2021 | 2021 | USA | **PV068290.1** |
| Gamma-P.1 | HTI/Haiti-4-66/2021 | 2021 | Haiti | **PP667486.1** |
| Gamma-P.1 | INEI104260 | 2021 | Argentina | **OZ061800.1** |

SARS-CoV-2, severe acute respiratory syndrome coronavirus 2
